# Influence of Auscultation on Practical Medicine

**Published:** 1842-07

**Authors:** 


INFLUENCE OF AUSCULTATION ON PRACTICAL MEDICINE.

We hope our readers will not suffer the length of Dr. Pope's translation to deter them from reading it; it will richly repay them for an attentive perusal. At all events it will enable them to answer the question so frequently asked: what good has auscultation done? The whole essay, of which the present number contains about one-half, strikes us as being a learned and ingenious one. As an evidence of its merit, we may mention that the Bordeaux Medical Society not only awarded to the author, Dr. Peyraud, its prize, but, as a further mark of distinction, conferred upon him the title of Corresponding Member. We shall endeavor to publish the remainder in our next.

Dr. Pope has recently returned from Paris, having spent two or three years in the assiduous observation of disease in the hospitals, and in attendance on the lectures of the distinguished men with whom that capital abounds. He has taken up his abode at St. Louis, where we learn he is rapidly advancing in public confidence. We spent part of our novitiate with Dr. P. and know him intimately. If a high and honorable ambition, joined to fine talents and an industry that knows no obstacles, can insure success, no man will meet with a greater measure than he. C.

